# Effect of Resveratrol, a Dietary-Derived Polyphenol, on the Oxidative Stress and Polyol Pathway in the Lens of Rats with Streptozotocin-Induced Diabetes

**DOI:** 10.3390/nu10101423

**Published:** 2018-10-04

**Authors:** Lech Sedlak, Weronika Wojnar, Maria Zych, Dorota Wyględowska-Promieńska, Ewa Mrukwa-Kominek, Ilona Kaczmarczyk-Sedlak

**Affiliations:** 1 Department of Ophthalmology, University Clinical Center, Medical University of Silesia in Katowice, 40-514 Katowice, Poland; wpdorota@gmail.com (D.W.-P.); emrowka@poczta.onet.pl (E.M.-K.); 2 Department of Ophthalmology, School of Medicine in Katowice, Medical University of Silesia in Katowice, 40-514 Katowice, Poland; 3 Department of Pharmacognosy and Phytochemistry, School of Pharmacy with the Division of Laboratory Medicine in Sosnowiec, Medical University of Silesia in Katowice, 41-200 Sosnowiec, Poland; wwojnar@sum.edu.pl (W.W.); mzych@sum.edu.pl (M.Z.); isedlak@sum.edu.pl (I.K.-S.)

**Keywords:** diabetes, oxidative stress, polyol pathway, rats, resveratrol, streptozotocin, lens

## Abstract

Resveratrol is found in grapes, apples, blueberries, mulberries, peanuts, pistachios, plums and red wine. Resveratrol has been shown to possess antioxidative activity and a variety of preventive effects in models of many diseases. The aim of the study was to investigate if this substance may counteract the oxidative stress and polyol pathway in the lens of diabetic rats. The study was conducted on the rats with streptozotocin-induced type 1 diabetes. After the administration of resveratrol (10 and 20 mg/kg *po* for 4 weeks), the oxidative stress markers in the lens were evaluated: activity of superoxide dismutase, catalase and glutathione peroxidase, as well as levels of total and soluble protein, level of glutathione, vitamin C, calcium, sulfhydryl group, advanced oxidation protein products, malonyldialdehyde, Total Oxidant Status and Total Antioxidant Reactivity. The obtained results indicate that the administration of resveratrol to the diabetic rats shows antioxidative properties. It is not a result of antiglycaemic activity but resveratrol probably directly affects the antioxidative system. Resveratrol did not affect the polyol pathway in the lens of diabetic rats. Our results may indirectly indicate benefits of consumption of foods as well as dietary supplements containing resveratrol in diminishing oxidative stress in lenses of individuals suffering from diabetes mellitus.

## 1. Introduction

Resveratrol (3,4′,5-trihydroxy-stilbene) is a polyphenolic compound discovered in 1939. The first part of its name ‘res’ means that this compound is a derivative of resorcinol (benzene-1,3-diol) and the ‘veratrol’ part indicates the white hellebore—*Veratrum grandiflorum* (Maxim. ex Miq.) O.Loes.—the plant in which roots resveratrol was found for the first time. Resveratrol occurs in many plants such as apples, blueberries, mulberries, peanuts, pistachios, plums, raspberries and soy. The highest concentrations of this stilbene were found in the dried roots and rhizomes of Japanese knotweed—*Reynoutria japonica* Houtt. synonyms *Fallopia japonica* (Houtt.) Ronse Decr., *Polygonum cuspidatum* Siebold & Zucc.—which are used in a form of tea in Traditional Chinese Medicine. In Europe, its main sources are dark varieties of grapes (*Vitis vinifera* L.) and red wines, containing roughly 3- to 10-fold more resveratrol than white grape varieties or white wines. Resveratrol can be synthesized in larger amounts by plants in response to pathogens and abiotic stress [1].

It has been shown, in both experimental and clinical studies, that resveratrol reveals many beneficial effects. It may prevent cardiovascular diseases, neurological disorders, diabetes, obesity, non-alcoholic fatty liver disease, lung dysfunction in asthma, aging and cancer. Moreover, it also has a favourable effect on bone homeostasis and skeletal muscle atrophy [1,2]. There are also reports describing the potential role of resveratrol in prevention or treatment of some ocular impairments such as age-related macular degeneration, cataract, glaucoma, diabetic retinopathy, thyroid eye disease or retinopathy of prematurity. It may also inhibit the growth of uveal melanoma or retinoblastoma. The positive effect of this stilbene on the eye structures is connected mostly with its anti-oxidative, anti-apoptotic, anti-inflammatory, anti-angiogenic and vasodilatative activities [3,4,5,6,7,8,9,10,11,12].

The scientific literature indicates that there is a link between diabetes mellitus, oxidative stress and cataract formation. Long-term hyperglycaemia leads to overproduction of reactive oxygen species (ROS) in mitochondria and in results in the imbalance between ROS and endogenous defence mechanisms. Protein oxidation in the lens leads to an accumulation of insoluble aggregates and light scattering by lens opacity [13,14,15]. In diabetic patients, there is a higher risk of cataract development than in healthy people of the same age. What is more, cataract is more likely to develop earlier in diabetic patients than in healthy people [15]. There is also a higher complication rate in diabetic patients undergoing cataract surgery [16].

Resveratrol is proven to be a potent antioxidant. Its antiradical and antioxidative activity results from its structure. Antioxidative properties of polyphenols depend mainly on the redox properties of the hydroxyl groups in phenolic moieties and the potential for electron delocalization across their chemical structure. In resveratrol’s structure, there are two phenolic rings: monophenol and diphenol, while an abstraction of a hydrogen atom from monophenolic hydroxyl group occurs rather easily. Moreover, resveratrol also has three hydroxyl groups, which play important role in radical scavenging, since it was reported, that antioxidant activity increases along with the number of –OH groups. These hydroxyl groups also help resveratrol to chelate metals, which is also an important feature in the prevention of ROS generation [17].

As hyperglycaemia may result in the development of cataract via oxidative stress, it seems to be reasonable to examine, whether dietary antioxidants may be helpful in the prevention or delaying of its onset. Therefore, the main goal of this study was to investigate if resveratrol administered orally at the doses of 10 and 20 mg/kg affects oxidative-stress related changes in the lens of diabetic rats.

## 2. Materials and Methods

### 2.1. Experiment Design, Animals and Diabetes Induction

The study was conducted with the approval of the Local Ethics Committee in Katowice, Poland (approval no. 36/2015).

Mature (3-month-old) Wistar male rats were provided by the Centre of Experimental Medicine at the Medical University of Silesia. The rats were divided into 4 experimental groups (*n* = 8–9): C—control, healthy rats, D—control diabetic rats, D+R10—diabetic rats treated orally with resveratrol at a dose of 10 mg/kg and D+R20—diabetic rats treated orally with resveratrol at a dose of 20 mg/kg. In the D, D+R10 and D+R20 groups of rats, diabetes was induced by a single intraperitoneal injection of streptozotocin (60 mg/kg) [18,19]. Streptozotocin was prepared as a solution in 0.1 M citric buffer, pH = 4.5. The C group received a single injection only with citric buffer. 14 days after streptozotocin injection, blood glucose level was measured with the MicroDot glucometer. The rats in which blood glucose level exceeded 200 mg/dL were classified to the further stage of the experiment. Resveratrol suspension in water was administered orally for 4 weeks to the D+R10 and D+R20 groups via intragastric tube, once a day. The rats in the C and D groups received only water. To ensure the proper dose of resveratrol, the rats were weighted at the whole time of the experiment. Moreover, body mass was recorded at the day of STZ injection (initial body mass), after 2 weeks from STZ injection (start body mass) and after 4 weeks of resveratrol and water administration (final body mass). The difference between final and start body mass represents body mass gain.

After 4 weeks of resveratrol administration, the rats were euthanized by injection of ketamine + xylazine mixture and cardiac exsanguination. The collected blood was centrifuged in order to obtain the serum. In the serum, the glucose and fructosamine levels were assessed. The lenses were extracted from the eyeball, weighted, then homogenized in the PBS buffer (10% *v*/*w*). In the total homogenate, the total protein level and malondialdehyde level was assayed. Remaining homogenate was centrifuged (4 °C, 15 min, 10,000× *g*), the supernatant was collected, divided into parts and frozen until use.

### 2.2. Glucose and Fructosamine Concentration in the Serum

The assessment of serum glucose and fructosamine concentrations was carried out using the commercially available kits (Glucose kit no. 11504, Fructosamine kit no. 11043; BioSystems S.A., Barcelona, Spain).

### 2.3. Enzymes and Sugars Related to Polyol Pathway in the Lens

Aldose reductase (AR) activity was evaluated according to the procedure described by Patel et al.: phosphate buffer (pH 6.2) and NADPH solution were added to the lens homogenates. The reaction was initiated by addition of d,l-glyceraldehyde. The plate was read for 5 min at 340 nm [20]. Sorbitol dehydrogenase activity was analysed using QuantiChrom™ Sorbitol Dehydrogenase Assay Kit (kit no. DSDH-100; BioAssay Systems, Hayward, CA, USA). Glucose and fructose concentrations were assessed using BioSystems kits (Glucose kit no. 11504, Fructose kit no. 23794) and sorbitol concentration was measured with EnzyChrom™ Sorbitol Assay Kit (kit no. ESBT-100; BioAssay Systems).

### 2.4. Total and Soluble Protein in the Lens

Estimation of total and soluble protein content was carried out as described in Lowry [21]: the mixture of 2% Na_2_CO_3_ solution in 0.1 M NaOH, 1% CuSO_4_ and 2% potassium-sodium tartrate was added to the aliquot of the homogenate. After 10 min, the Folin-Ciocalteu reagent was added and after 30 min of incubation, 200 µL of the mixture was transferred into the 96-well plate, then read at 750 nm. The standard curve was prepared with bovine serum albumin.

### 2.5. Advanced Glycation End Products and Total Sulfhydryl Groups in the Lens

Evaluation of advanced glycation end products (AGEs) content was conducted with OxiSelect™ Competitive ELISA Kit (Cell Biolabs, Inc., San Diego, CA, USA). Total sulfhydryl groups (-SH groups) content was estimated basing the protocol described by Ellmann: a phosphate buffer (pH 8.0) and DTNB reagent were added to the samples and after colour development, the reaction was read at 412 nm. Obtained results were calculated with the use of extinction coefficient = 13,600/M/cm [22].

### 2.6. Enzymatic Oxidative Stress Parameters in the Lens

Superoxide dismutase (SOD), catalase (CAT) and glutathione peroxidase (GPx) activity was assayed using Cayman kits (SOD kit no. 706002, CAT kit no. 707002. GPx kit no. 703102; Cayman Chemicals, Ann Arbor, MI, USA).

### 2.7. Non-Enzymatic Oxidative Stress Parameters Content in the Lens

Reduced glutathione (GSH) content was measured according to the method described by Sedlak and Lindsay: the homogenates 10% trichloroacetic acid was added to the homogenates in order to deprotein the samples. After centrifuging, the obtained supernatants were collected and transferred to the 96-well plate, then the phosphate buffer (pH 8.0) and 0.01 M DTNB were added. GSH standard was used as a reference. The reaction was measured at 412 nm [23]. Vitamin C level was assessed basing the Jagota and Dani method, in which trichloroacetic acid is used to deprotein the samples and acidify the reaction environment. In low pH, Folin-Ciocalteu reagent reacts specifically with vitamin C [24]. Advanced oxidation protein products (AOPP) estimation was conducted according to the protocol described by Witko-Sarsat et al. 1.16 M solution of potassium iodide and glacial acetic acid were added to the samples. The samples were read at 340 nm with chloramine-T used as a reference [25]. As a marker for lipid peroxidation malondialdehyde (MDA) was examined as described in Ohkawa et al. To the probes following reagents were added: 8.1% SDS, 20% acetic acid and 0.8% TBA. After thorough mixing, the mixture was heated for 60 min in boiling water. Afterwards, the probes were cooled and the mixture of pyridine with n-butanol was added. Probes were mixed and centrifuged at 4000× *g* for 5 min. Obtained supernatants were transferred to the 96-well plate and measured at 532 nm with 1,1,3,3-tetraethoxypropane used as a reference [26]. Calcium level was estimated with the Pointe Sci. kit (kit no. C7503, Pointe Scientific, Canton, MI, USA).

### 2.8. Total Oxidant Status and Total Antioxidant Response in the Lens

Total Oxidant Status (TOS) was assayed as described by Erel: to the probes the reagent 1 (consisted of mixture of 150 μM xylenol orange, mM NaCl 140 and 1.35 M glycerol in 25 mM H_2_SO_4_) and reagent 2 (5 mM ferrous ion and 10 mM o-dianisidine in 25 mM H_2_SO_4_) were added. The first absorbance (at 560 nm and 800 nm as reference) was taken before the mixing of reagents 1 and 2 and the last absorbance was taken after 4 min of mixing probes with reagent 2. The standard curve was prepared with H_2_O_2_ [27]. Total Antioxidant Response (TAR) estimation was conducted following the Erel protocol: the probes were mixed with reagent 1 (10 mM o-dianisidine, 45 μM ferrous ion in the 75 mM Clark and Lubs solution, pH 1.8) and with reagent 2 (7.5 mM H_2_O_2_ in the Clark and Lubs solution). Similarly like in TOS method, the first absorbance (at 444 nm) was taken before the mixing of reagents 1 and 2 and the last absorbance was taken after 4 min of mixing probes with reagent 2. The standard curve was prepared with Trolox [28]. Results obtained from TOS and TAR were used to calculate the TAR/TOS ratio.

### 2.9. Results Analysis

All measurements were conducted in microplate reader Tecan Infinite M200 PRO with Magellan 7.2 Software. Obtained results were subjected to statistical analysis with one-way ANOVA followed with Tukey post-hoc test in Statistica 10 Software (StatSoft, Tulsa, OK, USA). All results are presented as arithmetical mean ± SEM. Results were considered statistically significant if *p*-value < 0.05.

## 3. Results

### 3.1. Effect of Resveratrol on the Body Mass and Lens Mass in Diabetic Rats

In the non-diabetic animals (C group), the initial body mass was 281.2 ± 3.8 g. After 2 weeks of the experiment, the body mass (start body mass) was 312.8 ± 5.9 g. After the following 4 weeks, the final body mass in C rats was 344.9 ± 5.5 g. In the control diabetic rats (D group), 2 weeks after the administration of streptozotocin (STZ), the body mass (start body mass) was lower (*p* < 0.001) in comparison with the result obtained in C rats. The final body mass (after a further 4 weeks) was lower (*p* < 0.001) when compared to the C rats. In the diabetic rats receiving resveratrol at a dose of 10 mg/kg (D+R10) and 20 mg/kg (D+R20), the final body mass was similar to that observed in the D rats (Table 1).

The mass of the lens in the D, D+R10 and D+R20 groups was significantly lower (*p* < 0.001) as compared to the C group. No significant changes were noted in D+R10 or D+R20 groups when compared to the D group (Table 1).

### 3.2. Effect of Resveratrol on the Blood Glucose Concentration and Blood Fructosamine Concentration

Before STZ injection, the blood glucose concentration in all groups of rats was between 103 and 141 mg/dL (below 200 mg/dL). 2 weeks after STZ injection, the blood glucose concentration in the D, D+R10 and D+R20 groups exceeded the 200 mg/dL concentration and even reached values above 600 mg/dL. After 4 weeks of resveratrol administration, there were no significant changes with regard to this parameter in the D+R10 and D+R20 groups (Table 1).

In comparison with the C rats, in the D rats an increase of the fructosamine concentration in blood (*p* < 0.001) was observed. No statistically significant changes between D+R10, D+R20 and D groups were noted (Table 1).

### 3.3. Effect of Resveratrol on Polyol Pathway in the Lens of the Diabetic Rats

In the D rats, the lens glucose concentration was higher (*p* < 0.001), in comparison with the C rats. In the diabetic rats receiving resveratrol, the lens glucose concentration was similar to that observed in the D rats (Table 2).

The sorbitol and the fructose concentration in the lens was higher (*p* < 0.001 and *p* < 0.001, respectively) in the lens of the D rats, as compared to the C rats. No significant changes were observed in the sorbitol and fructose concentration after administration of resveratrol in diabetic rats (D+R10, D+R20 groups) when compared to the D rats (Table 2).

There was an increase of the aldose reductase activity and the sorbitol dehydrogenase activity in the lens of the D rats (*p* < 0.05) in comparison with the C rats. In the D+R10 and D+R20 groups, the activity of these enzymes was similar to those observed in the D rats (Table 2).

### 3.4. Effect of Resveratrol on the Advanced Glycation End Products (AGEs) Content and Sulfhydryl Group (-SH Groups) Content in the Lens of the Diabetic Rats

In comparison with the C rats, in the D rats an increase of the AGEs content in the lens was observed (*p* < 0.001). No significant changes were observed in the AGEs content after administration of resveratrol in diabetic rats (D+R10, D+R20 groups) when compared to the D rats (Table 2).

The -SH groups content in the lens of the D rats was lower, as compared to the C rats (not statistically significant). In the diabetic rats which received resveratrol (D+R10, D+R20 groups), the -SH groups content was similar to that observed in the C rats. When compared to the D group, in the D+R10 and D+R20 groups, the -SH groups content was elevated. The changes were not statistically significant (Table 2).

### 3.5. The Effect of Resveratrol on the Total and Soluble Protein Content in the Lens of the Diabetic Rats

No significant changes were observed in the total protein content after diabetes induction in rats (D group) as compared to the C rats. In the diabetic rats which received resveratrol (D+R10 and D+R20 groups), the total protein content was similar to that observed in the D and C rats (Table 3).

In the D group, the soluble protein content in the lens was lower (*p* < 0.05) in comparison with the C rats. The administration of resveratrol to the diabetic rats resulted in an increase of the soluble protein content in the D+R10 group (*p* < 0.05) and in the D+R20 group (not statistically significant) when compared to the D rats, while no changes in this parameter were recorded in comparison with the C group (Table 3).

### 3.6. Effect of Resveratrol on the Enzymatic Oxidative Stress Parameters in the Lens of the Diabetic Rats

**Figure 1:**
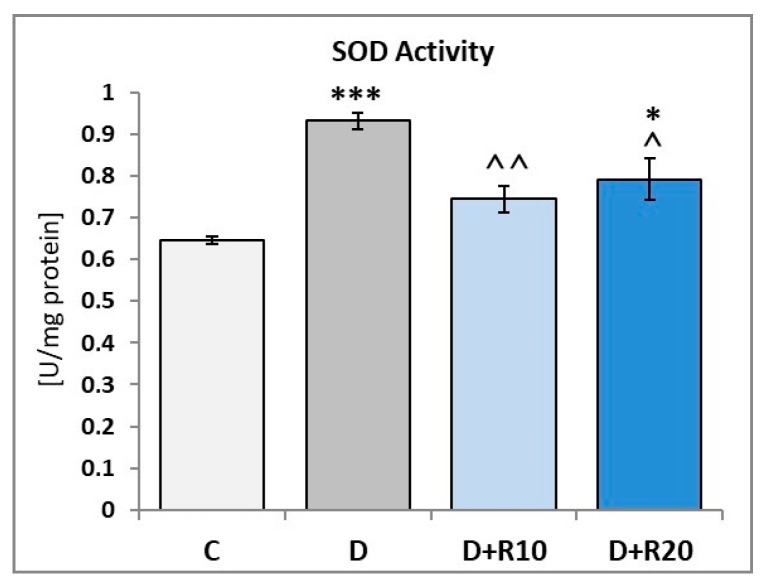
Effect of resveratrol administration on the superoxide dismutase (SOD) activity in the lens of the diabetic rats. C—control, non-diabetic rats; D—diabetic rats; D+R10—diabetic rats receiving resveratrol (10 mg/kg *po* for 4 weeks); D+R20—diabetic rats receiving resveratrol (20 mg/kg *po* for 4 weeks). Results are presented as means ± SEM (*n* = 8–9). * *p* < 0.05, *** *p* < 0.001—statistically significantly different from the C group. ^ *p* < 0.05, ^^ *p* < 0.01—statistically significantly different from the D group.

The SOD activity in the lens of the D rats was higher (*p* < 0.001) in comparison with the C rats. When compared to the C group, an activity of the SOD was elevated in the D+R10 group and D+R20 group (*p* < 0.05) but administration of resveratrol at both the doses led to a decrease (*p* < 0.01 and *p* < 0.05, respectively) in this parameter as compared to the D group (Figure 1).

**Figure 2:**
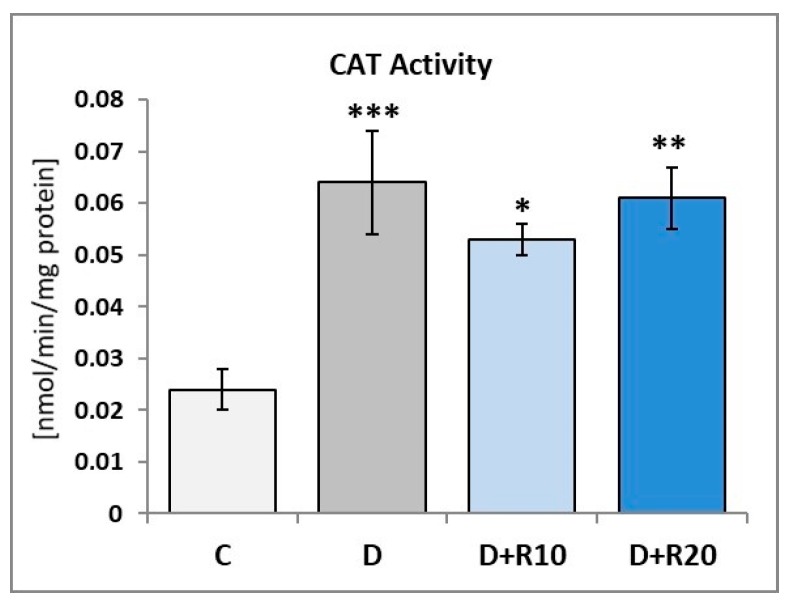
Effect of resveratrol administration on the catalase (CAT) activity in the lens of the diabetic rats. C—control, non-diabetic rats; D—diabetic rats; D+R10—diabetic rats receiving resveratrol (10 mg/kg *po* for 4 weeks); D+R20—diabetic rats receiving resveratrol (20 mg/kg *po* for 4 weeks). Results are presented as means ± SEM (*n* = 8–9). * *p* < 0.05, ** *p* < 0.01, *** *p* < 0.001—statistically significantly different from the C group.

In comparison with the C rats, in the D rats an increase of the CAT activity in the lens was observed (*p* < 0.001). In the D+R10 and D+R20 groups, there was a decrease of the CAT activity when compared to the D rats (not statistically significant) and an increase in this parameter when compared to the C rats (*p* < 0.05 and *p* < 0.01, respectively) (Figure 2).

**Figure 3:**
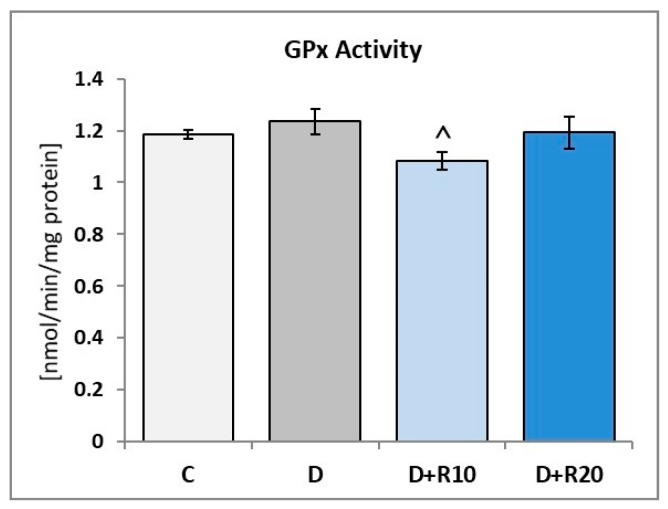
Effect of resveratrol administration on the glutathione peroxidase (GPx) activity in the lens of the diabetic rats. C—control, non-diabetic rats; D—diabetic rats; D+R10—diabetic rats receiving resveratrol (10 mg/kg *po* for 4 weeks); D+R20—diabetic rats receiving resveratrol (20 mg/kg *po* for 4 weeks). Results are presented as means ± SEM (*n* = 8–9). ^ *p* < 0.05 – statistically significantly different from the D group.

The activity of GPx in the lens was insignificantly higher in the D rats than in the C rats. When compared to the D group, in the D+R10 and D+R20 groups, activity of the GPx was decreased (*p* < 0.05 in D+R10). No statistically significant changes between D+R10, D+R20 and C groups were noted (Figure 3).

### 3.7. Effect of Resveratrol on the Non-Enzymatic Oxidative Stress Parameters Content in the Lens of the Diabetic Rats

In the lens of the D rats the content of GSH and vitamin C was lower (*p* < 0.001 and *p* < 0.05, respectively) in comparison with the C rats. Administration of resveratrol to the diabetic rats did not affect these parameters when compared to the D rats (Table 4).

In the lens of the D rats, the content of calcium was higher (*p* < 0.05) in comparison with the C rats. Administration of resveratrol at both doses did not affect the content of calcium when compared to the D rats. In comparison with the C group, in the D+R10 and D+R20 groups, this parameter was higher (*p* < 0.01 and not statistically significant, respectively) (Table 4).

**Figure 4:**
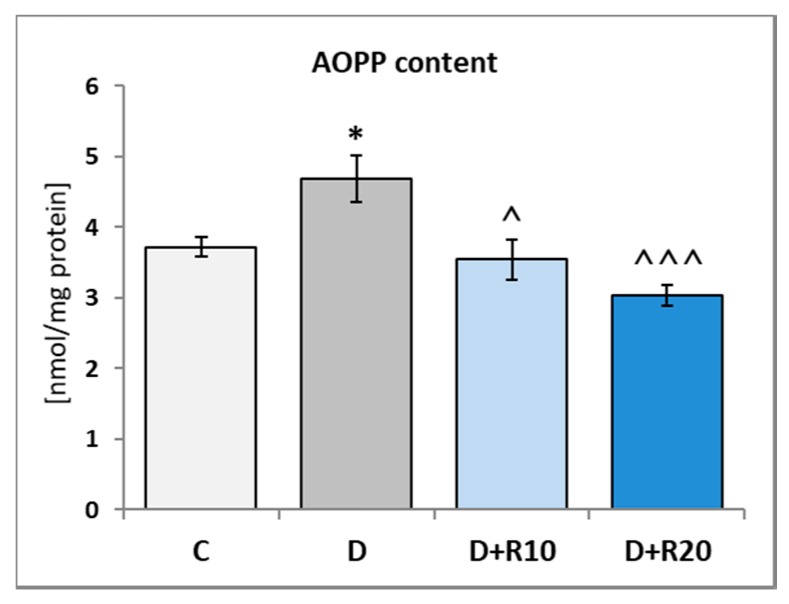
Effect of resveratrol administration on the advanced oxidation protein products (AOPP) content in the lens of the diabetic rats. C—control, non-diabetic rats; D—diabetic rats; D+R10—diabetic rats receiving resveratrol (10 mg/kg *po* for 4 weeks); DR20—diabetic rats receiving resveratrol (20 mg/kg *po* for 4 weeks). Results are presented as means ± SEM (*n* = 8–9). * *p* < 0.05—statistically significantly different from the C group. ^ *p* < 0.05, ^^^ *p* < 0.001—statistically significantly different from the D group.

In the D rats the AOPP content in the lens was higher (*p* < 0.05) as compared to the C rats. In comparison with the D rats, in the D+R10 and D+R20 groups, this parameter was lower (*p* < 0.05 and *p* < 0.001, respectively) and lower in comparison with the C group too (not statistically significant) (Figure 4).

**Figure 5:**
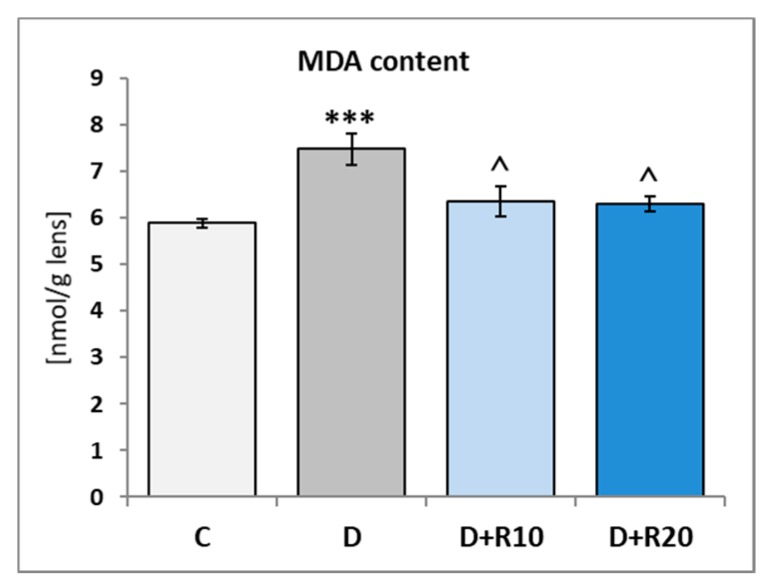
Effect of resveratrol administration on the malondialdehyde (MDA) content in the lens of the diabetic rats. C—control, non-diabetic rats; D—diabetic rats; D+R10—diabetic rats receiving resveratrol (10 mg/kg *po* for 4 weeks); D+R20—diabetic rats receiving resveratrol (20 mg/kg *po* for 4 weeks). Results are presented as means ± SEM (*n* = 8–9). *** *p* < 0.001—statistically significantly different from the C group. ^ *p* < 0.05—statistically significantly different from the D group.

The MDA content was higher (*p* < 0.001) in the lens of the D rats than in the C rats. Administration of resveratrol at both doses resulted in a decrease of the MDA content (*p* < 0.05) when confronted with the D group. In comparison with the C rats, in the D+R10 and D+R20 groups, this parameter was higher (not statistically significant) (Figure 5).

### 3.8. Effect of Resveratrol on the Total Antioxidant Reactivity (TAR) Total Antioxidant Reactivity (TAR) and Total Oxidant Status (TOS) in the Lens of the Diabetic Rats

**Figure 6:**
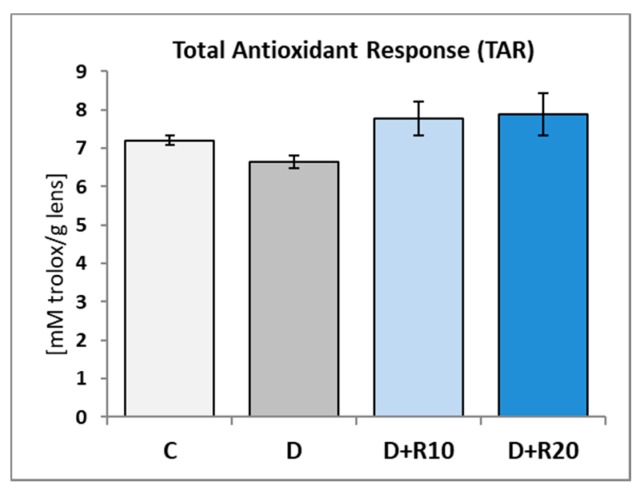
Effect of resveratrol administration on the Total Antioxidant Response (TAR) in the lens of the diabetic rats. C—control, non-diabetic rats; D—diabetic rats; D+R10—diabetic rats receiving resveratrol (10 mg/kg *po* for 4 weeks); D+R20—diabetic rats receiving resveratrol (20 mg/kg *po* for 4 weeks). Results are presented as means ± SEM (*n* = 8–9).

In the D rats the TAR in the lens was lower as compared to the C rats (not statistically significant). In comparison with the D rats, in the D+R10 and D+R20 groups, this parameter was higher and higher in comparison with the C group too (not statistically significant) (Figure 6).

**Figure 7:**
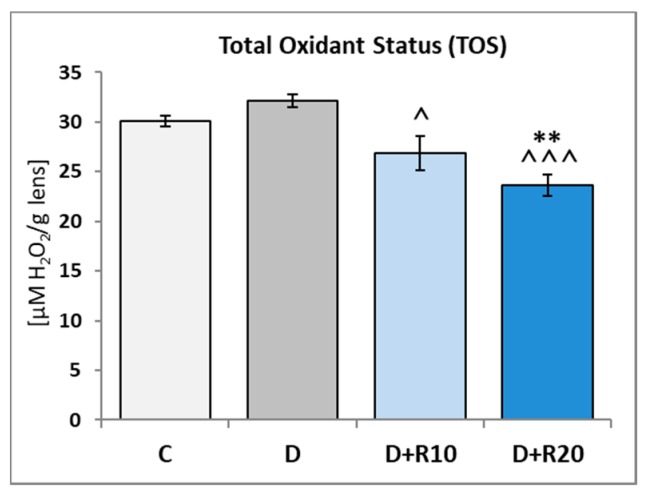
Effect of resveratrol administration on the Total Oxidant Status (TOS) in the lens of the diabetic rats. C—control, non-diabetic rats; D—diabetic rats; D+R10—diabetic rats receiving resveratrol (10 mg/kg *po* for 4 weeks); D+R20—diabetic rats receiving resveratrol (20 mg/kg *po* for 4 weeks). Results are presented as means ± SEM (*n* = 8–9). ** *p* < 0.01—statistically significantly different from the C group. ^ *p* < 0.05, ^^^ *p* < 0.001—statistically significantly different from the D group.

The TOS in the lens of the D rats was not statistically significantly higher, as compared to the C rats. When compared to the C group, in the D+R10 group and D+R20 group (*p* < 0.01), TOS was reduced and administration of resveratrol at both doses led to the decrease (*p* < 0.05 and *p* < 0.001, respectively) in this parameter, when compared to the D group (Figure 7).

**Figure 8:**
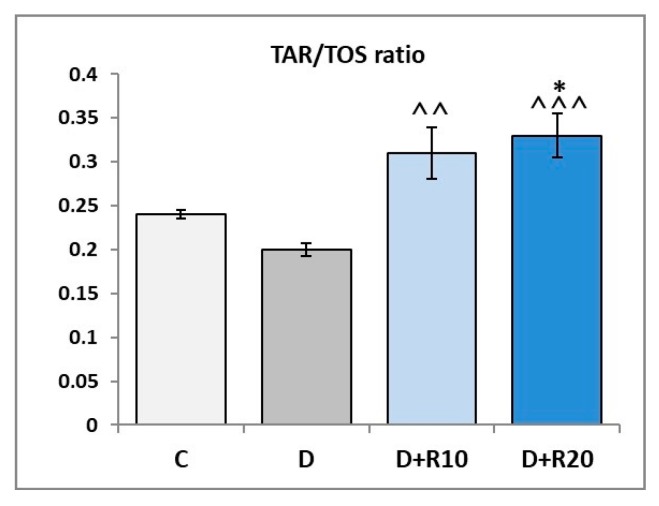
Effect of resveratrol administration on the TAR/TOS ratio in the lens of the diabetic rats. C—control, non-diabetic rats; D—diabetic rats; D+R10—diabetic rats receiving resveratrol (10 mg/kg *po* for 4 weeks); D+R20—diabetic rats receiving resveratrol (20 mg/kg *po* for 4 weeks). Results are presented as means ± SEM (*n* = 8–9). * *p* < 0.05—statistically significantly different from the C group. ^^ *p* < 0.01, ^^^ *p* < 0.001—statistically significantly different from the D group.

The TAR/TOS ratio in the lens was not statistically significantly lower in the D rats than in the C rats. When compared to the C group, in the D+R10 and D+R20 groups TAR/TOS ratio was elevated (not statistically significantly and *p* < 0.05, respectively). Administration of resveratrol at both doses resulted in an increase of the TAR/TOS ratio (*p* < 0.01 and *p* < 0.001, respectively), when confronted with the D group (Figure 8).

No statistically significant changes between the D+R10 and D+R20 group in the all parameters were reported.

## 4. Discussion

Diabetes mellitus (DM) is a disease characterized by chronic hyperglycaemia and disturbances of carbohydrate, fat and protein metabolism resulting from an absolute or relative deficiency of insulin. Increased oxidative stress is thought to play an important role in the pathogenesis of diabetic complications, as supported by increased levels of oxidized DNA, proteins and lipids. The induction of oxidative stress in DM can result from multiple mechanisms both non-enzymatic and enzymatic mechanisms.

In our study, the standard model of type 1 diabetes induced by streptozotocin was used [18,19]. We have noted, that an induction of type 1 diabetes in rats resulted in an increased blood glucose concentrations, blood fructosamine concentrations, lens glucose concentration and a decrease in the body mass of rats. This result overlaps with other studies conducted in diabetic rats [29,30,31].

It is known, that high glucose levels can stimulate oxidative stress by auto-oxidation of glucose and via non-enzymatic process of advanced glycation end products (AGEs) formation. We observed an increase of AGEs in the lens of rats with STZ-induced type 1 diabetes. An elevated lens AGEs level in diabetic rats was also described elsewhere [29,32]. Scientific evidences indicate that accumulation of AGEs in lens leads to accelerated cataractogenesis in hyperglycaemic experimental animals as well as in humans [33,34].

Another important mechanism whereby high glucose levels can induce oxidative stress is the polyol pathway. In our study, activation of the polyol pathway results in an increase of the sorbitol and fructose content in the lens of diabetic. This fact may be linked to an elevated glucose concentration in this tissue. Excessive accumulation of sorbitol increases osmotic stress, along with cross-linking with proteins of the lens by non-enzymatic glycosylation, promoting the formation of high molecular weight insoluble proteins which are responsible for the loss of transparency of the lens [35,36]. We have also noted an increased activity of polyol pathway enzymes—sorbitol dehydrogenase and aldose reductase.

We assume that lower body mass of rats with diabetes is associated with lower lens mass. These results also overlap with previous reports [30,31,37]. In the present study, the reason of the decrease in the lens mass was not a protein impoverishment because the total content of protein in the lens of the diabetic rats was the same as that in the non-diabetic rats. However, induction of diabetes in rats caused a decrease of content of soluble protein in the lens. This observation is confirmed by other studies [30,31]. Crystallins, soluble proteins of the lens, are important for the maintenance of lens transparency and the prevention of cataracts.

In the course of diabetes, the formation of protein aggregates in lens results from a decrease of sulfhydryl groups content in protein [38]. Similarly, in our study, we report a decrease of sulfhydryl groups content in the lens of rats with diabetes.

It has been well established that an increase of AOPP content is related to numerous different pathological states, underlined directly by oxidative stress, including diabetes [34]. AOPP content has increased in the lens in our study.

There are several reports in the literature demonstrating the elevated content of malondialdehyde (final products of polyunsaturated fatty acids peroxidation) in the lens [31] and other tissues [39,40,41,42,43,44,45,46] in STZ-induced diabetes in rats. The results of the present study are also in line with the previous reports.

Endogenous antioxidative mechanisms include both enzymatic and non-enzymatic processes. Two antioxidants (GSH and vitamin C) are fundamentally important for scavenging and correcting any damage due to ROS in the lens [47]. We observed, that GSH content and vitamin C content were reduced in the lens of diabetic rats. This result overlaps with other studies conducted on the lens of diabetic rats [29,30,31].

Some of the principal components of the oxidative defence system include SOD, CAT and GPx. Numerous previous studies indicate that changes in the activity of these enzymes are one of the major causes of diabetic cataractogenesis due to the imbalance between free radicals and antioxidants [35]. In the present study, oxidative stress in the lens of diabetic rats intensified antioxidant response by an increase of activity of SOD, CAT and GPx. These findings are in agreement with previous studies in diabetic animal [30,31,35,37].

Proper lens calcium concentration provides transparency of this tissue and its increase is associated with both human and animal lens opacification [29,48]. Likewise, in our study, we noted an elevated calcium concentration in the lens of diabetic rats.

In this study, the presence of oxidative stress in the diabetic lens was evidenced by the increased TOS and a decrease in TAR together with a reduction in TAR/TOS ratio. Many researchers have confirmed the changes of parameters of oxidative stress in the lens in the animal model of diabetes, thus indicating the contribution of ROS in the pathogenesis of lens opacity [35,49,50].

Resveratrol, as a dietary stilbene, is present in many plant-derived products (products made from grapes, soybeans, apples, blueberries, mulberries, peanuts, pistachios, plums, raspberries) and dietary supplement. In the present study, we examined, for the first time, the effect of orally administered resveratrol at the doses of 10 and 20 mg/kg on parameters of oxidative stress in vivo in the lens of the rats with STZ-induced type 1 diabetes. No changes in action after using both doses of resveratrol were observed. Doses of resveratrol were chosen based on the literature [38,42,44,45,51,52,53,54].

Administration of resveratrol did not influence the body mass and blood glucose and fructosamine concentrations of the diabetic rats. Likewise, the use of resveratrol did not change an increased glucose and fructose concentration in the lens of diabetic rats. Sorbitol content, as well as the activity of sorbitol dehydrogenase and aldose reductase associated with polyol pathway in lens, were not altered by resveratrol.

Since no effect on body mass, blood glucose concentration, blood fructosamine concentration, glucose and fructose concentration in the lens, which was observed after resveratrol administration, it can be presumed, that this stilbene shows no strict antidiabetic effect in this experimental model. Varsha et al., Ates et al., Schmatz et al., Yu et al., Bagatini et al., Faid et al. have documented that resveratrol administered to rats with streptozotocin-induced type 1 diabetes does not cause changes in blood glucose concentrations and does not affect the body mass [29,39,42,43,51,55]. Other studies have demonstrated that resveratrol decreases blood glucose in animals with hyperglycaemia [40,44,45,54]. Similarly, ambiguous results are obtained in humans [2,56,57].

However, despite the lack of effect of resveratrol on hyperglycaemia in our studies, we have observed that this compound shows other beneficial effects in the lens of diabetic rats. Soluble protein content and the sulfhydryl groups content were increased in the lens. To the best of our knowledge, there is only one study of influence on sulfhydryl groups content after administration of resveratrol. Asadi et al. reported an increase of sulfhydryl groups content in the serum after the use of resveratrol at the dose of 10 mg/kg for 30 days in rats with diabetes [38].

Furthermore, administration of resveratrol resulted in favourable changes in oxidative stress parameters in the lens of the streptozotocin-induced diabetic rats. The studied substance also normalizes an increased activity of basic enzymes taking part in oxidative stress id est SOD, CAT and GPx. In diabetic rats, resveratrol used in doses from 5 to 20 mg/kg demonstrated beneficial effect on SOD and CAT activity in blood [38], aorta [54], kidney [42,45], brain [44,46], corpus cavernosus penis [43] and liver [42,54].

These findings are also confirmed by other oxidative stress-related parameters which were assessed in the lens—AOPP and MDA content. Both parameters increased by diabetes induction and decreased after administration of resveratrol. Doganay et al. triggered oxidative stress and cataract in rat pups by injection of sodium selenite subcutaneously. Afterwards, the animals have been receiving resveratrol for 21 days (40 mg/kg intraperitoneal). The researchers observed that resveratrol suppressed lens opacification in rats. This protective effect was supported by lower MDA content in lens [6]. In sugar-induced lens opacity ex vivo model, resveratrol showed a significant protective effect preventing a decrease in transparency and formation of polyols in cattle lens [32]. Numerous scientific reports indicate that MDA level decreases under the influence of resveratrol in different tissues of diabetic rats: brain [39,41,44,46], liver [42,52,54], kidneys [40,42,45,53], corpus cavernosus penis [43].

Resveratrol does not influence GSH content in diabetic rats in our study. This is in line with observations made by Sadi and Konat [41], who did not find an effect of resveratrol on GSH content in the brain of diabetic rats.

In our study, we confirmed an indirect evidence for the antioxidant effect of resveratrol in the diabetic lens by noting a significant decrease of TOS, as well as an increase of TAR and TOS/TAR ratio. Administration of resveratrol to diabetic rats decreased TOS in the brain [41], blood [38], liver [52] and increased TAR in the brain [52]. Among the different beneficial effects of resveratrol found in diabetes, the ability of this compound to reduce oxidative stress seems to be best documented.

It can be concluded from our study that resveratrol shows antioxidative properties also in the lens of diabetic rats. It is not a result of antiglycaemic activity but resveratrol probably directly affects the antioxidative system. This conclusion might be confirmed by a fact of increasing defence antioxidant system by resveratrol in different tissues of healthy animals. Venturini et al. demonstrated that treatment with resveratrol administered to healthy rats at the dose of 20 mg for 21 days increases antioxidant capacities in the brain of healthy rats (an increase of SOD activity in striatum and CAT activity in the hippocampus, a decrease of MDA level in the striatum and frontal cortex) [46]. Faid et al. also reported a raised antioxidant potential in testes of healthy animals as an effect of the increased activity of SOD, CAT, GPx [55]. In another study, administration of resveratrol resulted in an increase in antioxidant defence system, namely SOD and CAT activity and a decrease of MDA level in the liver of normal rats [58].

Our results may indirectly indicate benefits of consumption of foods as well as dietary supplements containing resveratrol in diminishing oxidative stress in lenses of individuals suffering from diabetes mellitus. It requires further research.

## Figures and Tables

**Table 1:** Effect of resveratrol on the body mass, lens mass, blood glucose concentration and blood fructosamine concentration in diabetic rats.

Parameter/Group	C	D	D+R10	D+R20
Initial body mass (g)	281.2 ± 3.8	283.2 ± 6.2	282.0 ± 3.7	276.6 ± 5.5
Start body mass (g)	312.8 ± 5.9	250.2 ± 8.7 ***	239.4 ± 4.9 ***	242.8 ± 6.6 ***
Final body mass (g)	344.9 ± 5.5	228.2 ± 10.9 ***	212.2 ± 5.7 ***	228.1 ± 7.4 ***
Body mass gain (g)	32.1 ± 2.8	−22.0 ± 5.1 ***	−27.2 ± 4.9 ***	−14.7 ± 3.5 ***
Lens mass (mg)	47.2 ± 0.5	42.9 ± 1.7 ***	42.8 ± 0.4 ***	43.7 ± 0.5 ***
Glucose in the blood (mg/dL)	141.4 ± 11.0	641.9 ± 28.6 ***	613.9 ± 55.5 ***	641.7 ± 51.6 ***
Fructosamine in the blood (µmol/L albumin)	275.1 ± 8.1	498.4 ± 21.3 ***	489.7 ± 19.4 ***	471.5 ± 54.7 **

C—control, non-diabetic rats; D—diabetic rats; D+R10—diabetic rats receiving resveratrol (10 mg/kg *po* for 4 weeks); D+R20—diabetic rats receiving resveratrol (20 mg/kg *po* for 4 weeks). Results are presented as means ± SEM (*n* = 8–9). ** *p* < 0.01, *** *p* < 0.001—statistically significantly different from the C group.

**Table 2:** Effect of resveratrol on polyol pathway and the advanced glycation end products (AGEs) content and sulfhydryl groups (-SH groups) content in lens in diabetic rats.

Parameter/Group	C	D	D+R10	D+R20
Glucose in the lens (mg/g lens)	0.16 ± 0.03	0.83 ± 0.07 ***	1.02 ± 1.00 ***	0.80 ± 0.07 ***
Sorbitol (µmol/g lens)	1.26 ± 0.05	30.10 ± 0.47 ***	29.42 ± 0.70 ***	30.73 ± 0.66 ***
Fructose in the lens (µmol/g lens)	0.08 ± 0.01	0.13 ± 0.01 ***	0.14 ± 0.01 ***	0.13 ± 0.01 ***
Aldose reductase (nmol/min/mg protein)	0.085 ± 0.003	0.107 ± 0.008 *	0.102 ± 0.003 **	0.109 ± 0.005 *
Sorbitol dehydrogenase (µU/mg protein)	1.94 ± 0.18	3.01 ± 0.34 *	2.33 ± 0.14	2.67 ± 0.15 *
AGEs (µg/g lens)	5.80 ± 0.32	10.17 ± 0.29 ***	10.30 ± 0.12 ***	9.94 ± 0.50 ***
-SH groups (nmol/g lens)	3.63 ± 0.13	3.09 ± 0.15	3.38 ± 0.20	3.91 ± 0.34

C—control, non-diabetic rats; D—diabetic rats; D+R10—diabetic rats receiving resveratrol (10 mg/kg *po* for 4 weeks); D+R20—diabetic rats receiving resveratrol (20 mg/kg *po* for 4 weeks). Results are presented as means ± SEM (*n* = 8–9). * *p* < 0.05, ** *p* < 0.01, *** *p* < 0.001—statistically significantly different from the C group.

**Table 3:** Effect of resveratrol on the total and soluble protein content in the lens in diabetic rats.

Parameter/Group	C	D	D+R10	D+R20
Total protein (mg/g lens)	563.2 ± 33.2	536.4 ± 43.6	511.5 ± 13.4	559.8 ± 26.9
Soluble protein (mg/g lens)	443.8 ± 8.4	408.5 ± 13.9 *	453.5 ± 8.6 ^	428.0 ± 12.0

C—control, non-diabetic rats; D—diabetic rats; D+R10—diabetic rats receiving resveratrol (10 mg/kg *po* for 4 weeks); D+R20—diabetic rats receiving resveratrol (20 mg/kg *po* for 4 weeks). Results are presented as means ± SEM (*n* = 8–9). * *p* < 0.05 – statistically significantly different from the C group. ^ *p* < 0.05 – statistically significantly different from the D group.

**Table 4:** Effect of resveratrol on the glutathione (GSH), vitamin C and calcium content in the lens in diabetic rats.

Parameter/Group	C	D	D+R10	D+R20
GSH (µmol/g lens)	4.10 ± 0.61	0.78 ± 0.13 ***	0.75 ± 0.11 ***	0.74 ± 0.10 ***
Vitamin C (µg/g lens)	7.69 ± 0.05	7.17 ± 0.02 *	7.20 ± 0.02 *	7.21 ± 0.06 *
Calcium (µg/g lens)	42.6 ± 2.1	53.8 ± 3.1 *	60.3 ± 2.5 **	50.4 ± 4.9

C—control, non-diabetic rats; D—diabetic rats; D+R10—diabetic rats receiving resveratrol (10 mg/kg *po* for 4 weeks); D+R20—diabetic rats receiving resveratrol (20 mg/kg *po* for 4 weeks). Results are presented as means ± SEM (*n* = 8–9). * *p* < 0.05, ** *p* < 0.01, *** *p* < 0.001 – statistically significantly different from the C group.
